# Family Structure as a Correlate of Organized Sport Participation among Youth

**DOI:** 10.1371/journal.pone.0147403

**Published:** 2016-02-10

**Authors:** Rachel McMillan, Michael McIsaac, Ian Janssen

**Affiliations:** 1 Department of Public Health Sciences, Queen’s University, Kingston, Ontario, Canada; 2 School of Kinesiology and Health Studies, Queen’s University, Kingston, Ontario, Canada; Instituto de Higiene e Medicina Tropical, PORTUGAL

## Abstract

Organized sport is one way that youth participate in physical activity. There are disparities in organized sport participation by family-related factors. The purpose of this study was to determine whether non-traditional family structure and physical custody arrangements are associated with organized sport participation in youth, and if so whether this relationship is mediated by socioeconomic status. Data were from the 2009–10 Health Behaviour in School-aged Children survey, a nationally representative cross-section of Canadian youth in grades 6–10 (N = 21,201). Information on family structure was derived from three survey items that asked participants the number of adults they lived with, their relationship to these adults, and if applicable, how often they visited another parent outside their home. Participants were asked whether or not they were currently involved in an organized sport. Logistic regression was used to compare the odds of organized sport participation according to family structure. Bootstrap-based mediation analysis was used to assess mediation by perceived family wealth. The results indicated that by comparison to traditional families, boys and girls from reconstituted families with irregular visitation of a second parent, reconstituted families with regular visitation of a second parent, single-parent families with irregular visitation of a second parent, and single-parent families with regular visitation of a second parent were less likely to participate in organized sport than those from traditional families, with odds ratios ranging from 0.48 (95% confidence interval: 0.38–0.61) to 0.78 (95% confidence interval: 0.56–1.08). The relationship between family structure and organized sport was significantly mediated by perceived family wealth, although the magnitude of the mediation was modest (ie, <20% change in effect estimate). In conclusion, youth living in both single-parent and reconstituted families experienced significant disparities in organized sport participation that was partially mediated by perceived family wealth.

## Introduction

Physical inactivity is associated with decreased mental and physical health in children and youth [1]. It is therefore concerning that children and youth are becoming less active and fit [2]. Organized sport offers one way for young people to engage in physical activity [3]. Participation is associated with improved health and decreased engagement in several risk behaviours, including illegal drug use and excessive screen time [3–5]. Furthermore, youth who participate in sport are more likely to be physically active as adults, allowing them to reap lifelong health benefits including decreased all-cause mortality [6]. It is therefore beneficial to identify determinants of youths’ organized sport participation.

Family structure may be one such determinant. Today, 32% of Canadian youth live in non-traditional families such as a single-parent family or reconstituted family, which includes a stepparent or parent’s partner [7]. There is some evidence linking a non-traditional family structure with organized sport participation. For instance, results from the 2010 General Social Survey in Canada showed that 74% of 5–14 year olds from dual-parent families had participated in organized sport during the past year, which is modestly higher than the 68% participation rate observed in 5–14 year olds from single-parent families [8]. Several other studies have concluded that youth from single-parent families are less likely to participate in sport and that this relationship may be moderated by the child’s gender [9–11], although null findings were observed in one study [12]. Many of these studies were limited by their use of a simple single- or dual-parent definition of family structure. This ignores potential differences between traditional dual-parent families and reconstituted dual-parent families. It also fails to take into account how shared custody or visitation with a non-residential parent may influence organized sport participation. This type of visitation is associated with improvements in some of the child health outcomes that are also related to organized sport [13, 14].

The pathway(s) that explains the association between family structure and organized sport participation remains unclear. This association may be explained in part by less favourable socioeconomic status (SES) conditions in youth from non-traditional families [15], which may influence their ability to participate in health-related behaviours such as organized sport [16]. Indeed, the proportion of 5–19 year olds who participate in organized sport decreases with decreasing household income [17].

The primary purpose of this study was to examine whether participation in organized sport (yes or no) differed in youth from traditional dual-parent families, single-parent families, and reconstituted families, while also considering the effects of visitation with the non-custodial parent. A secondary purpose was to evaluate whether perceived family wealth as an indicator of SES was a mediator of this relationship. This research could potentially inform future targeted interventions aimed at reducing disparities in organized sport participation among youth.

## Materials and Methods

### Study Design and Population

Study data are from the nationally representative cross-sectional 2009–10 Canadian Health Behaviour in School-aged Children Survey (HBSC). The HBSC is conducted every four years in 43 countries in collaboration with the World Health Organization [18]. The present study is limited to the Canadian data. The HBSC consists of a standardized self-report survey filled out in a classroom setting, with the goal of determining the prevalence and distribution of a wide range of psychological, social and physical determinants of health in 11–15 year olds. All HBSC questionnaire items are continuously developed, piloted and validated by the HBSC international network [18].

The 2009–10 Canadian HBSC received ethics approval from Health Canada and the General Research Ethics Board at Queen’s University. Consent was provided by school boards, individual schools, student participants, and their parents/guardians. The study consisted of 26,068 students in grades 6–10 from 436 public schools across Canada. All provinces and territories participated, with the exceptions of New Brunswick and Prince Edward Island, who chose not to participate. Combined, these two provinces represent less than 3% of the Canadian population.

The student response rate was 77%. The provincial samples were obtained using a two-tiered cluster-sampling procedure to sample entire classrooms for participation, while all students living in the three territories were invited to participate if they met the study inclusion criteria in order to ensure adequate representation. All students from selected classrooms were eligible and invited to participate. Students attending private, on-reserve, special needs or home-based schools were excluded, as were those who did not provide consent for participation or who were absent from school on the day the survey was completed.

**Fig 1:**
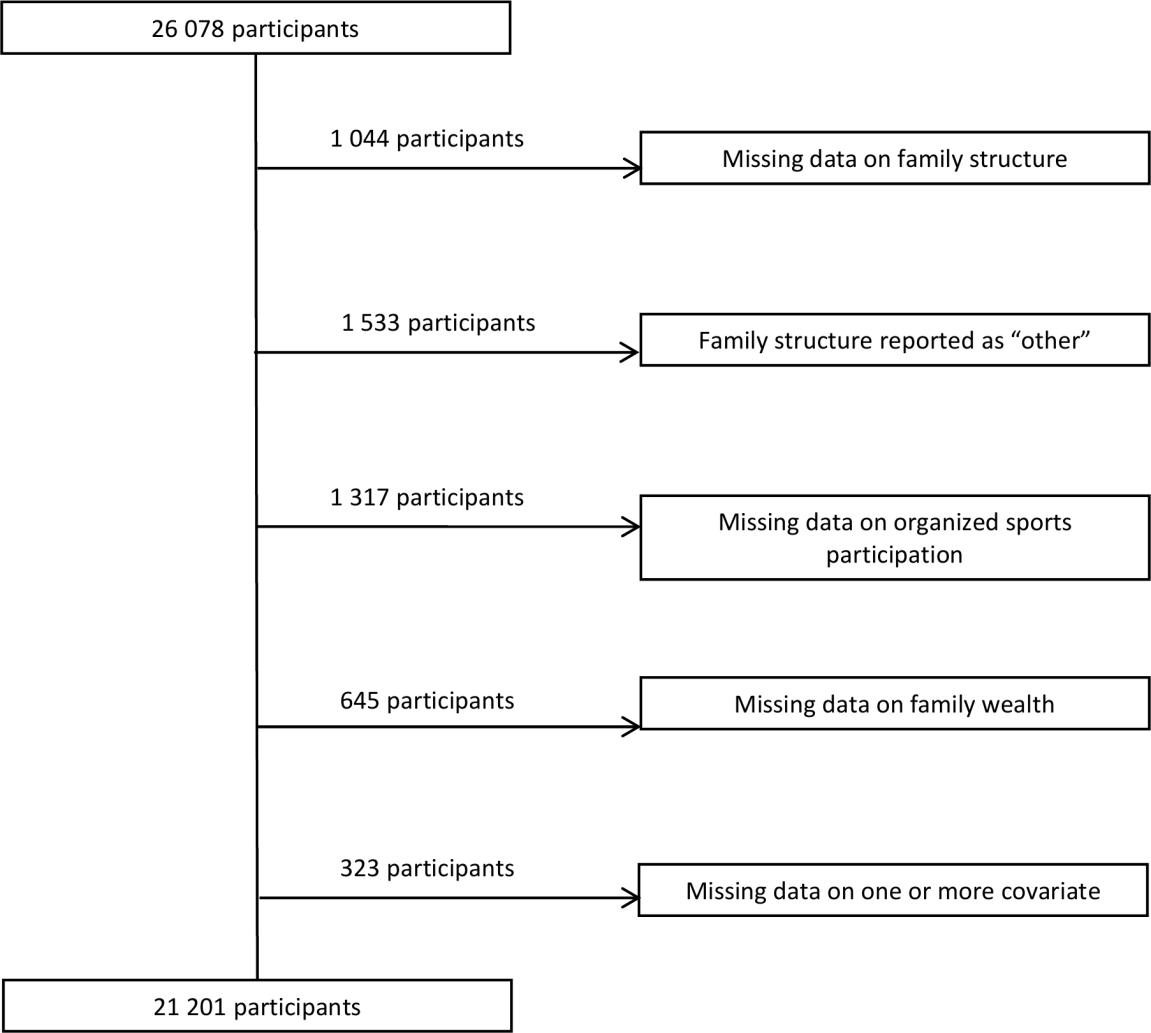
Flow chart of inclusion information for participants.

Participants were included in the analysis if they had complete data for all of the questions of interest and lived with at least one of their parents. A total of 4,877 participants were excluded from the analysis for the reasons outlined in Fig 1. This left a final sample of 21,201 participants. Descriptive characteristics of excluded participants were similar to included participants (data not shown).

### Exposure (Family Structure)

Information on family structure was derived from three questions. The first asked participants to check off the adults who live in the home *“where [they] live all or most of the time”* from a list of choices including mother, father, stepmother (or father’s girlfriend) and stepfather (or mother’s boyfriend). The second asked whether they had a second home. Participants who responded “yes” to this question were then asked how often they stayed in their second home (“*half the time*”, “*regularly but less than half the time*”, “*sometimes*” or “*hardly ever*”). Based on responses to these questions, participants were defined as living in a traditional family (includes both a mother and a father), a single-parent family (includes either a mother or a father), or a reconstituted family (includes either a mother or a father and either a stepmother/father’s girlfriend or stepfather/mother’s boyfriend. Youth from non-traditional families were further defined as having “regular visitation” with a second parent if they had a second home and reported visiting it “*half the time*” or “*regularly but less than half the time*” and “irregular visitation” if the they reported not having a second home, or having a second home but visiting it “*sometimes*” or “*hardly ever*”. Youth who reported that neither their mother nor their father lived in their primary home constituted ~4% of the sample and were excluded from the analyses.

### Outcome (Organized Sport Participation)

Organized sport participation was assessed by a question that asked participants whether they were involved in any “*sport club or team*”, with two response options (“*yes”* or “*no”*). A study of the 2-week test-retest reliability of a similarly worded question from the American Youth Risk Behavior Survey showed that students in grades 7 and 10 reliably reported their organized sport participation over the past year (*r* = 0.84) [19].

### Covariates

Potential covariates were selected based on previous literature and their availability within the HBSC. These included gender, grade, ethnicity (Canadian, which includes those who self-identified as Caucasian or Aboriginal; East and Southeast Asian; South Asian; Black; Arab; or other, which includes those of mixed ethnicity and those who self-identified as other), immigration status (“*born in Canada/immigrated >5 years*” or “*immigrated ≤5 years*”), and presence of siblings in the primary home (“*yes*” or “*no*”).

### Socioeconomic Status as a Mediator

Perceived family wealth was used as an indicator of SES. It was assessed using a single item on the HBSC that asked participants to report *“how well off [they] think [their] family is”*, with five ordinal responses ranging from “*not at all well off”* to *“very well off”*. This variable was treated as a continuous variable during regression analyses.

### Statistical Analysis

All analyses used survey procedures in SAS 9.4 to account for the complex sampling design used by the HBSC, including clustering and sampling weights. All analyses were stratified by gender as it has been shown previously that sports participation levels as well as reasons for participation differ by gender [20]. The HBSC sample was characterized using simple descriptive statistics.

**Fig 2:**
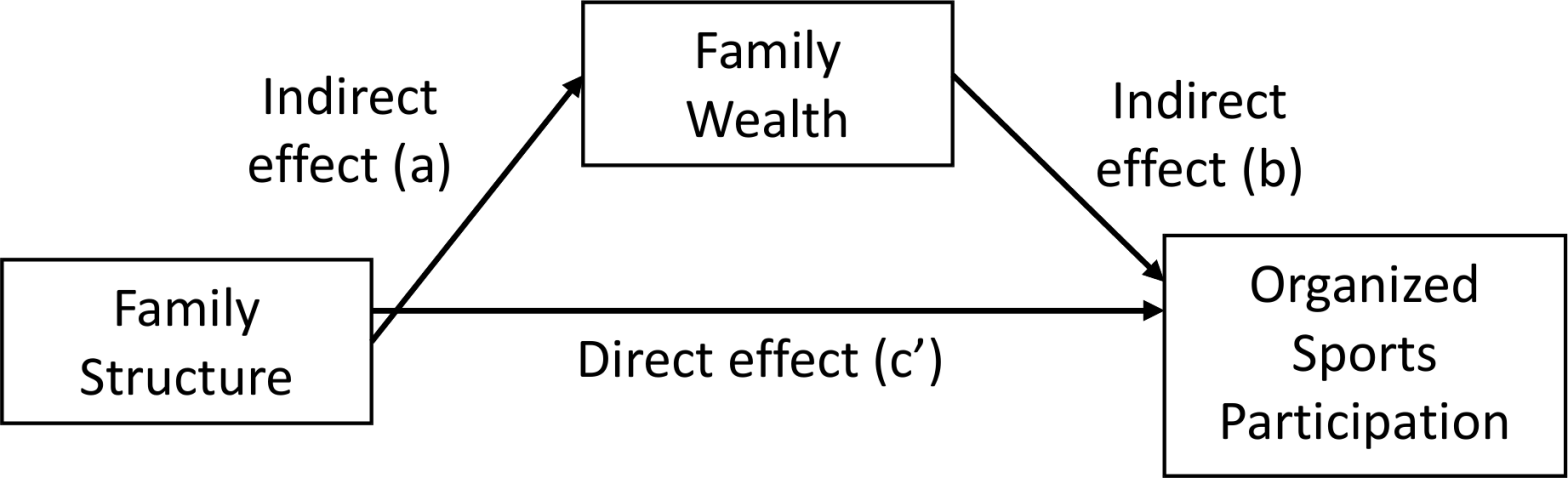
The direct effect and indirect effect of family structure on organized sport participation, considering family affluence as a mediator.

A contemporary mediation analysis approach was used to assess the total, direct and indirect associations of family structure on organized sport participation, considering perceived family wealth as a potential mediator of this relationship [21, 22]. These associations are depicted in Fig 2. The total association represents the full effect of the exposure, family structure, on the outcome, organized sport. The mediator, perceived family wealth, represents an intermediate factor that falls on the causal pathway between the exposure and the outcome [21, 22]. If mediation is present, all (full mediation) or part (partial mediation) of the total effect is transmitted on the outcome through the mediator. The direct association represents the portion of the total effect that occurs independently of the pathway through the proposed mediator, perceived family wealth (path *c’* in Fig 2). The indirect association, on the other hand, is the portion of the total effect that can be accounted for by family structure’s effect on perceived family wealth (path *a* in Fig 2), which in turn affects organized sport participation (path *b* in Fig 2).

Multiple logistic regression was used to quantify the total association between family structure and organized sport participation after controlling for covariates, without adjusting for perceived family wealth (see *c’* pathway in Fig 2), as well as the direct association, which did adjust for perceived family wealth. The covariates considered in these analyses were gender, grade, ethnicity, immigration status, and presence of siblings in the primary home. Final covariate selection for the multivariate models was performed through backwards deletion using a 10% change-in-estimate threshold [23]. If a covariate changed the odds ratio of at least one non-traditional family structure by more than 10% for either boys or girls in any of the total or indirect models of sports participation, it was included in all final models. All covariates of interest met this criterion.

The indirect association between family structure and organized sport (see the *a* and *b* pathways in Fig 2) was estimated using a bootstrap sampling procedure with 2500 resamples, controlling for relevant covariates identified in the previous analysis. This was done in SAS using a modified version of a macro developed by Carson and colleagues [24]. The *a* pathways were estimated for each family structure category through multiple linear regression (SAS Proc SurveyReg), while the *b* pathways were estimated using multiple logistic regression (SAS Proc SurveyLogistic) for each of the resamples. Point estimates of the indirect associations and their 95% bootstrap-based confidence intervals were calculated for each pathway based on the products of the two regression coefficients for each resample. There was evidence of mediation if the 95% confidence interval did not include 0, the null value.

## Results

### Sample Characteristics

Demographic characteristics of the participants are in Table 1. The majority were of Canadian ethnicity and considered their families to be “quite well off” or “very well off”. Almost three quarters lived in traditional families. Approximately 55% participated in organized sport.

**Table 1:** Descriptive Characteristics of Participants.

Variable	N	% (95% CI)*
**Gender**		
Male	10 157	47.8 (46.2, 49.4)
Female	11 044	52.2 (50.6, 53.8)
**Grade**		
Grade 5	39	0.2 (0.0, 0.5)
Grade 6	3 950	18.4 (15.0, 21.7)
Grade 7	4 113	19.5 (17.0, 21.9)
Grade 8	4 348	20.8 (18.2, 23.4)
Grade 9	4 477	21.2 (17.9, 24.5)
Grade 10	4 152	19.5 (16.1, 22.8)
Grade 11	122	0.5 (0.3, 0.7)
**Perceived Family Wealth**		
Very well off	4 940	23.4 (22.2, 24.5)
Quite well off	6 889	34.2 (32.8, 35.5)
Average	7 409	33.4 (32.1, 34.8)
Not very well off	1 380	6.7 (6.1, 7.4)
Not at all well off	583	2.3 (2.0, 2.6)
**Immigrant Status**		
Lived in Canada ≥5 years	20 335	95.6 (94.7, 96.5)
Lived in Canada <5 years	866	4.4 (3.5, 5.3)
**Parental Structure**		
Traditional family	14 930	71.0 (69.6, 72.4)
Reconstituted with irregular visitation	1 586	7.1 (6.5, 7.7)
Reconstituted with regular visitation	607	3.0 (2.6, 3.3)
Single-parent with irregular visitation	3 184	14.3 (13.4, 15.3)
Single-parent with regular visitation	894	4.5 (4.1, 5.0)
**Siblings**		
≥1 sibling	18 171	86.9 (86.1, 87.7)
Only child	3 030	13.1 (12.3, 13.9)
**Ethnicity**		
Canadian	17 149	76.4 (73.0, 79.9)
East and Southeast Asian	1 102	5.9 (4.1, 7.7)
South Asian	561	3.3 (2.2, 4.4)
Black	347	2.3 (1.7, 2.9)
Arab	179	1.2 (0.7, 1.7)
Latin American	160	0.9 (0.6, 1.3)
Other	1 916	9.9 (8.9, 10.9)
**Participation in Sports Club or Team**		
No	9 298	44.9 (43.0, 46.7)
Yes	11 903	55.1 (53.3, 57.0)

N = Number of sampled individuals with complete valid data for all variables presented.

*Estimated population characteristics after adjusting for sampling weights and clustering by classroom, school and province.

Table 2 shows the proportion of boys and girls who participated in organized sport by family structure. Within both boys and girls, the proportion participating in organized sport was lower among non-traditional families than among traditional families. For youth from reconstituted and single-parent families, organized sport participation was higher in the regular visitation subgroup than the irregular visitation subgroup.

**Table 2:** Organized Sport Participation by Family Structure.

Family Structure	% Participate in Organized Sports (95% CI)*
**Boys**	
Traditional	59.3 (57.0, 61.5)
Reconstituted with irregular visitation	**41.1 (35.8, 46.4)**
Reconstituted with regular visitation	53.4 (46.2, 60.7)
Single-parent with irregular visitation	**44.9 (41.2, 48.6)**
Single-parent with regular visitation	50.9 (44.2, 57.6)
**Girls**	
Traditional	53.6 (51.3, 55.8)
Reconstituted with irregular visitation	**38.8 (33.9, 43.6)**
Reconstituted with regular visitation	**44.2 (38.1, 50.2)**
Single-parent with irregular visitation	**36.6 (32.9, 40.3)**
Single-parent with regular visitation	46.2 (40.2, 52.1)

All analyses were adjusted for sample weights and clustering.

*Proportions with 95% confidence intervals not overlapping those of traditional families are shown in **bold**.

### Association Between Family Structure and Family Wealth

Lower perceived family wealth scores were observed in non-traditional family structures (*p* < 0.0001, Table 3). When perceived family wealth was treated as a 5-point ordinal scale, youth from non-traditional families perceived their family wealth as being 0.18 to 0.49 units lower than youth from traditional families.

**Table 3:** Association Between Family Structure and Perceived Family Wealth.

Family Structure	Beta Regression Coefficient (Standard Error)*	
	Boys	Girls
Traditional	0 (referent)	0 (referent)
Reconstituted with irregular visitation	-0.31 (0.05)	-0.37 (0.05)
Reconstituted with regular visitation	-0.21 (0.07)	-0.18 (0.07)
Single-parent with irregular visitation	-0.44 (0.05)	-0.49 (0.04)
Single-parent with regular visitation	-0.33 (0.06)	-0.37 (0.05)

All analyses were adjusted for sample weights and clustering and the following covariates: number of siblings, immigration status, ethnicity, and grade.

*All non-traditional family structure groups were significantly different from the traditional group (*p* ≤ 0.01).

### Association Between Perceived Family Wealth and Organized Sport

After controlling for covariates, each one-unit increase in perceived family wealth was associated with a 22% (95% CI: 1.15–1.29) increase in the odds of participating in organized sport among boys and a 24% (95% CI: 0.17–1.31) increase in the odds of participating in organized sport among girls.

### Association Between Family Structure and Organized Sport

Total association. Table 4 shows the relative odds of organized sport participation for each of the non-traditional family structures compared to traditional families. Before accounting for the effects of perceived family wealth, boys and girls had significantly lower odds of participating in organized sport if they were from any of the non-traditional family structures (exception: reconstituted with regular visitation group in boys).

**Table 4:** Results of the analyses examining the association between family structure and organized sport participation and the extent to which this was mediated by perceived family wealth.

Family Structure	Total Association	Direct Association		Indirect Association Point Estimate
	Odds Ratio* (95% CI)	Odds Ratio^†^ (95% CI)	% Change from Total Association ^§^	(Percentile 95% CI)
**Boys (N = 10 157)**				
Traditional	1.00 (referent)	1.00 (referent)	*-*	0 (referent)
Reconstituted with irregular visitation	0.48 (0.38, 0.61)	0.51 (0.40 0.64)	4.4	-0.05 (-0.08, -0.03)
Reconstituted with regular visitation	0.78 (0.56, 1.08)	0.80 (0.58, 1.11)	11.7	-0.03 (-0.06, -0.01)
Single-parent with irregular visitation	0.58 (0.49, 0.69)	0.62 (0.53, 0.73)	10.0	-0.07 (-0.10, -0.04)
Single-parent with regular visitation	0.73 (0.56, 0.95)	0.77 (0.59, 1.00)	14.4	-0.05 (-0.08, -0.03)
**Girls (N = 11 044)**				
Traditional	1.00 (referent)	1.00 (referent)	*-*	0 (referent)
Reconstituted with irregular visitation	0.53 (0.43, 0.66)	0.57 (0.46, 0.70)	7.1	-0.06 (-0.09, -0.04)
Reconstituted with regular visitation	0.63 (0.48, 0.81)	0.64 (0.50, 0.83)	4.5	-0.03 (-0.06, -0.01)
Single-parent with irregular visitation	0.54 (0.46, 0.63)	0.58 (0.49, 0.69)	9.5	-0.08 (-0.11, -0.05)
Single-parent with regular visitation	0.72 (0.56, 0.93)	0.77 (0.59, 0.99)	16.5	-0.06 (-0.09, -0.04)

All analyses were adjusted for sample weights and clustering.

*Adjusted for number of siblings, immigration status, ethnicity, and grade.

^†^Adjusted for number of siblings, immigration status, ethnicity, grade, and perceived family wealth.

^§^ Percentage change in the odds ratio from Total Association to Direct Association model (i.e., prior to and after controlling for family wealth). Calculated as: (OR_unadjusted for wealth_−OR_adjusted for wealth_)/ (OR_unadjusted for wealth_− 1)

Direct association. Including perceived family wealth in the regression model consistently increased the odds of organized sport participation for non-traditional families, bringing them closer to the odds for traditional families (Table 4). By comparison to the odds ratios observed for the total associations, the changes in the magnitude of the odds ratios ranged from 4.4% to 16.5% which implies that 4.4% to 16.5% of the association between family structure and organized sport was mediated by perceived family wealth.

Indirect association. Table 4 shows the results of the test of mediation of the relationship between family structure and organized sport participation by perceived family wealth. There was statistical evidence of mediation for all non-traditional family structures. The bootstrap-based point estimates for the mediation analyses are equal to the products of coefficients generated through different forms of regression, and are therefore not directly meaningful [22]. As organized sport participation is a binary outcome, the range of the point estimates is -1 (complete negative mediation) to 1 (complete positive mediation). The indirect effect of non-traditional family structure on organized sport transmitted by perceived family wealth was negative. Most of the point estimates were small in magnitude (i.e., between -0.1 and 0), regardless of statistical significance.

## Discussion

We looked at differences in organized sport participation by family structure in a large and representative sample of Canadian youth. We also considered whether perceived family wealth was a mediator of this relationship. We found that youth from single-parent and reconstituted families were less likely to participate in organized sport than those from traditional families regardless of custody arrangements. Another major finding was that the relationship between family structure and organized sport participation was partially mediated by perceived family wealth.

The majority of previous studies looking at the relationship between family structure and organized sport participation defined family structure as simply single- or dual-parent. One of these studies showed that girls from single-parent families were less likely to participate in sport than girls from dual-parent families [3], two showed that this relationship between single- and dual-parent family structure and sports participation existed in boys but not in girls [9, 10], and one found no relationship in either gender [12]. A key finding of the current study was that organized sport participation differed between youth from traditional and non-traditional families, and that this difference was similar in magnitude regardless of whether the youth who lived in non-traditional families were from single-parent or reconstituted families.

It is unclear why there are differences in organized sport participation between traditional and reconstituted dual-parent families. One potential explanation is that stepparents may be less engaged in childcare than biological parents [25]. Another potential explanation is that the average SES is lower in reconstituted families than traditional families [25]. A final potential explanation is that youth from reconstituted dual-parent families are more likely to transition between single-parent and reconstituted families, such as when a single-parent remarries.

Some previous studies of the relationship between non-traditional family structure and youth organized sport participation controlled for SES as a covariate. Our study considered SES, as assessed by perceived family wealth, as a mediator on the causal pathway of the relationship between family structure and organized sport participation. Our findings suggested that there was weak-to-moderate mediation. Therefore, family wealth and other SES measures are likely only one of the mediating pathways through which family structure influences organized sport participation. Other plausible mediating pathways include family dynamics, access to sport facilities, parental support of sport-related behaviours, and availability of a co-parent to assist in transportation to and from organized sport activities.

It has been hypothesized that shared physical custody arrangements may lead to decreases in physical activity given that visiting a second non-residential parent may lead to logistical complications or inconsistencies in support for behaviours such as organized sport participation [26]. Our findings did not support this hypothesis as we found that youth who visited a non-residential parent regularly were not less likely to participate in organized sport than those from similar non-traditional family structures who rarely or never visited a non-residential parent. This finding may partially reflect the fact that non-residential parents who have regular visitation with their children are more likely to contribute financially to their care [27]. Indeed, the influence of mediation by family structure was marginally stronger for youth from non-traditional families who did not have regular visitation with their second parent.

As increasing numbers of youth are exposed to non-traditional family structures, it is becoming increasingly important to understand why disparities in health-related behaviours exist according to family structure and how best to intervene to diminish these disparities. The findings of this study suggests that interventions aimed at increasing organized sport participation in youth might be more successful if they consider both family structure and the financial cost of sports participation. This could be done by targeting non-traditional families with advertisements or information to increase their awareness of financial incentives to cover the cost of their children’s sports participation, such as the Canadian federal government’s Children’s Fitness Tax Credit [28]. In the case of low-income families unable to cover the up-front cost of organized sport participation, another more viable intervention might be for sports organizations to subsidize the cost of low-income youth participation and then apply for government funding equivalent to the tax credit. Another option for addressing disparities in organized sport participation by family structure might be to address the time constraints experienced by some single-parent families. Providing youth from such families with community- or school-based transportation to and from sporting events might help address this problem.

One strength of this study was its large sample size, which allowed us the statistical power to compare reconstituted and traditional families and also divide non-traditional families by custody arrangements. In addition, the HBSC is nationally representative and therefore generalizable to the Canadian youth population. A final strength is the use of a contemporary bootstrap-based test of mediation by perceived family wealth. This test is high in power compared to other methods of testing mediation [21, 22].

Our study also has some limitations. All data were based on self-report and are therefore subject to recall and reporting biases. The use of cross-sectional data meant that we were unable to determine how long participants had been in their current family structure or the timeframe of their organized sport participation, which may have led to exposure and outcome misclassification. We were also only able to consider organized sport participation as a yes or no dichotomous outcome, and future research could be enhanced by considering it as a continuum with different levels of participation. Several potential covariates and mediators, such as parental sports participation and employment status, were not available in the dataset. Furthermore, some of the covariates that were considered in the analyses, such as ethnicity and immigration status, had to be simplified into dichotomous variables for the mediation analysis. Finally, selection bias was a concern given that youth who did not provide consent or who were absent from school on the day of the survey may have been systematically different from those who did participate.

## Conclusion

Youth from single-parent and reconstituted families have lower odds of participating in organized sport than those from traditional families and that this relationship was partially mediated by SES disparities. Future interventions aimed at increasing organized sport participation in youth from non-traditional family structures should consider the financial cost of participation and consider approaches to increase parent’s awareness of financial incentives to cover these costs. Future research should focus on elucidating additional mediating pathways between family structure and sports participation.
